# Potent Activity of the HIV-1 Maturation Inhibitor Bevirimat in SCID-hu Thy/Liv Mice

**DOI:** 10.1371/journal.pone.0001251

**Published:** 2007-11-28

**Authors:** Cheryl A. Stoddart, Pheroze Joshi, Barbara Sloan, Jennifer C. Bare, Philip C. Smith, Graham P. Allaway, Carl T. Wild, David E. Martin

**Affiliations:** 1 Gladstone Institute of Virology and Immunology, University of California at San Francisco, San Francisco, California, United States of America; 2 School of Pharmacy, University of North Carolina, Chapel Hill, North Carolina, United States of America; 3 Panacos Pharmaceuticals, Gaithersburg, Maryland, United States of America; Federal University of Sao Paulo, Brazil

## Abstract

**Background:**

The HIV-1 maturation inhibitor, 3-*O*-(3′,3′-dimethylsuccinyl) betulinic acid (bevirimat, PA-457) is a promising drug candidate with 10 nM in vitro antiviral activity against multiple wild-type (WT) and drug-resistant HIV-1 isolates. Bevirimat has a novel mechanism of action, specifically inhibiting cleavage of spacer peptide 1 (SP1) from the C-terminus of capsid which results in defective core condensation.

**Methods and Findings:**

Oral administration of bevirimat to HIV-1-infected SCID-hu Thy/Liv mice reduced viral RNA by >2 log_10_ and protected immature and mature T cells from virus-mediated depletion. This activity was observed at plasma concentrations that are achievable in humans after oral dosing, and bevirimat was active up to 3 days after inoculation with both WT HIV-1 and an AZT-resistant HIV-1 clinical isolate. Consistent with its mechanism of action, bevirimat caused a dose-dependent inhibition of capsid-SP1 cleavage in HIV-1-infected human thymocytes obtained from these mice. HIV-1 NL4-3 with an alanine-to-valine substitution at the N-terminus of SP1 (SP1/A1V), which is resistant to bevirimat in vitro, was also resistant to bevirimat treatment in the mice, and SP1/AIV had replication and thymocyte kinetics similar to that of WT NL4-3 with no evidence of fitness impairment in in vivo competition assays. Interestingly, protease inhibitor-resistant HIV-1 with impaired capsid-SP1 cleavage was hypersensitive to bevirimat in vitro with a 50% inhibitory concentration 140 times lower than for WT HIV-1.

**Conclusions:**

These results support further clinical development of this first-in-class maturation inhibitor and confirm the usefulness of the SCID-hu Thy/Liv model for evaluation of in vivo antiretroviral efficacy, drug resistance, and viral fitness.

## Introduction

There remains an urgent need for more effective antiretrovirals to treat HIV/AIDS, in particular novel compounds that have activity against virus resistant to currently approved treatments. Drug resistance occurs in >50% of patients on antiretroviral therapy and is the leading cause of treatment failure [1]. The small-molecule inhibitor, 3-*O*-(3′,3′-dimethylsuccinyl) betulinic acid (bevirimat, formerly known as PA-457 or DSB) is the lead drug candidate in a recently described class of antiretrovirals termed maturation inhibitors [2], [3]. The drug holds great promise for specific and potent pharmacologic inhibition of HIV-1 replication. Here we report that orally administered bevirimat potently reduces viral load and prevents human T-cell depletion in a small animal model of HIV-1 infection.

Bevirimat targets a unique point of vulnerability in the HIV-1 life cycle: cleavage of the 14-amino-acid spacer peptide (SP1) from the capsid (CA) precursor (p25) in Pr55Gag by the viral protease, which is the final step required for maturation of the capsid and optimal virion infectivity [4], [5]. Recent structural studies indicate that CA hexamers polymerize to form a lattice within the mature HIV-1 virion [6], and it has been proposed by Vogt [7] that proteolytic maturation of Gag proteins prepares the virion for a new round on infection by destabilization of the virus core. This process facilitates CA dissociation in the next target cell and perhaps promotes reverse transcription of the viral genome. The bevirimat-mediated block in CA processing results in defective core condensation and the release of immature virus particles incapable of productive infection [2], [3], [8], [9]. Pharmacologic inhibition of CA-SP1 cleavage leading to virus particles with altered cores is thus analogous to the phenotype of CA point mutants with suboptimal core stability that are impaired at an early postentry step prior to reverse transcription [10]. Bevirimat is a triterpenoid derivative of betulinic acid, a compound with weak anti-HIV-1 activity isolated from *Syzigium claviforum*, an herb used in traditional Chinese medicine. When modified by the addition of a dimethyl succinyl moiety at the 3′-hydroxy position to yield bevirimat, 3-*O*-(3′,3′-dimethylsuccinyl) betulinic acid, anti-HIV-1 activity was increased >1,000 fold [11]–[14]. Bevirimat has potent [50% inhibition concentration (IC_50_) of ∼10 nM] antiviral activity against multiple wild-type (WT) and drug-resistant clinical HIV-1 isolates [2].

In confirmation of the mechanism of action of bevirimat, HIV-1 with an alanine-to-valine (A-to-V) substitution at the N-terminus of SP1 (and thus at the cleavage junction of CA-SP1) is resistant to bevirimat in vitro [2]. Further work has identified other in vitro resistance mutations at the C-terminus of CA and the N-terminus of SP1 [15], while point deletion mutagenesis showed that amino acids in the N-terminal half of SP1 serve as determinants of bevirimat activity [16]. Mutations at the CA-SP1 cleavage site conferring bevirimat resistance lead to impaired binding of the compound to Gag in immature HIV-1 virions during particle assembly [17].

Here we report potent antiviral activity of bevirimat in the SCID-hu Thy/Liv mouse model of HIV-1 infection, which we and others have shown to be highly reproducible and predictive for the evaluation of in vivo antiretroviral efficacy [18]–[20]. Twice-daily oral administration of bevirmat to HIV-1-infected SCID-hu Thy/Liv mice reduced implant viral loads in a dose-dependent manner, causing reductions of >2 log_10_ in HIV-1 RNA and ≥90% both in implant p24 concentration and in percentage of Gag-p24^+^ thymocytes at 100 mg/kg per day while preserving immature and mature T-cell populations. Antiviral activity was observed in the mice at plasma concentrations that are achievable in humans by oral dosing. HIV-1 with an A-to-V substitution at the N-terminus of SP1 is resistant to bevirimat treatment in the mice. In this animal model, the SP1/A1V mutant had viral replication and thymocyte depletion kinetics similar to WT HIV-1. At 28 days after inoculation, there was no evidence of reversion to WT and no detectable fitness impairment in in vivo competition assays. Our results confirm the usefulness of the SCID-hu Thy/Liv model for preclinical evaluation of in vivo antiretroviral efficacy, drug resistance, and viral fitness.

## Methods

### Viruses

The following reagent was obtained through the AIDS Research and Reference Reagent Program, Division of AIDS, NIAID, NIH: pNL4-3 from Malcolm Martin [21]. Bevirimat-resistant NL4-3 SP1/A1V (also designated as A364V based on the full Gag sequence) was supplied by Panacos Pharmaceuticals. Stocks of NL4-3 and SP1/A1V were prepared by transfection of 293T cells and collection of supernatants on days 2 or 3. The AZT-resistant (M41L, T215Y) R5X4 clinical HIV-1 isolate JD was kindly provided by Mike McCune and prepared by cocultivation of patient peripheral blood mononuclear cells (PBMCs) and expansion in phytohemagglutinin (PHA)-activated PBMCs. We introduced the I54V and V82A protease inhibitor resistance substitutions into the protease gene of HIV-1 NL4-3 by site-directed mutagenesis to produce NL4-3 PR_I54V+V82A_. Virus stocks were titrated by 50% endpoint assay in PHA-activated PBMCs with p24 detection by ELISA.

### SCID-hu Thy/Liv Mice

Human fetal thymus and liver were obtained through services provided by a nonprofit organization (Advanced Bioscience Resources) in accordance with federal, state, and local regulations. Coimplantation of thymus and liver fragments under the kidney capsule to create SCID-hu Thy/Liv mice and inoculation of the Thy/Liv implants with HIV-1 was carried out as described [18], [22]. Male C.B-17 SCID (model #CB17SC-M, homozygous, C.B-*Igh-1^b^*/IcrTac-Prkdc*^scid^*) mice were obtained at 6–8 weeks of age from Taconic, and cohorts of 50–60 SCID-hu Thy/Liv mice were implanted with tissues from a single donor. Implants were inoculated 18 weeks after implantation with 50 µl of stock virus (500–2,000 50% tissue-culture infectious doses) or RPMI 1640 medium (mock infection) by direct injection. Animal protocols were approved by the UCSF Institutional Animal Care and Use Committee. Groups of 5–8 mice each were treated with the N-methylglucamine di-salt of bevirimat at 10, 30, and 100 mg/kg per day (dosed as free-acid equivalents in 10% hydroxypropyl-β-cyclodextrin vehicle) by twice-daily oral gavage of 200 µl per dose.

### Implant Collection and Viral Load Quantification

The Thy/Liv implants were collected from euthanized mice, and single-cell suspensions were prepared by dispersing the implant through nylon mesh and processed for p24 ELISA, bDNA assay, and FACS analysis as described [18], [19], [23].

### Flow Cytometry

Implant cells were stained with phycoerythrin cyanine dye CY7-conjugated anti-CD4 (BD Biosciences), phycoerythrin cyanine CY5.5-conjugated anti-CD8 (Caltag), allophycocyanin cyanine CY7-conjugated anti-CD3 (eBiosciences), and phycoerythrin-conjugated anti-W6/32 (DakoCytomation). Cells were fixed and permeabilized with 1.2% paraformaldehyde and 0.5% Tween 20, stained with fluorescein isothiocyanate-conjugated anti-p24 (Beckman Coulter), and analyzed on an LSR II (BD Biosciences). After collecting 100,000 total cell events, percentages of marker-positive (CD4^+^, CD8^+^, and CD4^+^CD8^+^) thymocytes in the implant samples were determined by first gating on a live lymphoid cell population identified by forward- and side-scatter characteristics and then by CD3 expression.

### Western Blot Analysis of HIV-1-Infected Human Thymocytes

The SCID-hu Thy/Liv implants were collected 21 days after inoculation, disrupted into a single-cell suspension, and cultured in RPMI 1640 supplemented with 10% fetal bovine serum and 50 ng/ml IL-7 (growth medium) for 2 h. The dispersed Thy/Liv implant cells were checked for viability, equally distributed in fresh growth medium, and incubated for an additional 48 h in the presence of a range of concentrations of bevirimat free acid. The culture supernatant was collected, and cellular debris was removed by centrifugation at 1,000× g. Cell-free supernatants were loaded onto a 30% sucrose cushion, and virions were sedimented at 27,000× g for 2 h. Samples were normalized based on p24 levels, and the lysed virions were resolved on a 10–20% denaturing polyacrylamide gel. The separated viral proteins were then transferred to a polyvinylidene difluoride membrane, and individual viral proteins were visualized by Western blotting using anti-HIV-1 Ig (AIDS Reagent Program) and anti-human IgG peroxidase conjugate (Pierce). The efficiency of p25 cleavage in the presence of increasing concentrations of bevirimat was quantified using a molecular imager (BioRad).

To determine the effect of bevirimat on CA maturation of protease inhibitor-resistant HIV-1, Western blot analysis was performed on HIV-1 NL4-3 and NL4-3 PR_I54V+V82A_ virions collected from transfected 293T cells treated with 20 µM bevirimat as described above using a p24 specific monoclonal antibody (AIDS Reagent Program).

### RT-PCR and Sequencing of CA-SP1 RNA

Total RNA was extracted from frozen thymocyte pellets using Trizol LS (Invitrogen) and resuspended in nuclease-free water. The CA-SP1 region of the HIV-1 *gag* was amplified by RT-PCR using 10 µl of purified RNA and AmpliTaq Gold (Applied Biosystems) according to the manufacturer's instructions. PCR amplification was limited to 12–14 cycles, the PCR products were gel purified (Qiagen) and cloned into the pCR4-TOPO TA vector (Invitrogen), and DNA from randomly picked bacterial colonies (100–150 colonies per sample) was sequenced in both directions (Applied Biosystems). Nucleotide sequences of the amplified CA-SP1 region were aligned to the NL4-3 reference sequence (GenBank accession #M19921).

### In Vitro Antiviral Activity Assay

Pooled human PBMCs from six donors were thawed, cultured for 2 days RPMI 1640 supplemented with 10% fetal bovine serum, L-glutamine, and human lymphocyte IL-2, and inoculated in bulk at a multiplicity of infection of 0.005. Cells were washed, and 100,000 cells in 100 µl ml were added to triplicate wells of round-bottom 96-well plates. Wells were treated with 100 µl of serial half-log dilutions of bevirimat free acid or with medium alone, and supernatants were assayed at day 7 for p24 by ELISA. Parallel cellular toxicity determinations were performed by incubating bevirimat-treated uninfected PBMCs with (4,5-dimethylthiazol-2-yl)-2,5-diphenyl tetrasodiumbromide (MTT) on day 7. The 50% inhibitory concentration (IC_50_) and 50% cytotoxic concentration (CC_50_) were calculated using a 4-parameter curve fit (Softmax Pro, Molecular Devices).

### Analysis of Plasma Bevirimat Concentrations

Mouse plasma samples (20 µl) were diluted to 100 µl with 1% acetic acid (HAc). Aliquots of the dilutions (50 µl) and bevirimat serial standards spiked in blank mouse samples were protein-precipitated by adding 200 µl of ice-cold acetonitrile (ACN) followed by addition of an internal standard, 3-O-(3′,3′-dimethylsuccinyl)-dihydrobetulinic acid) (DSD, 100 ng). The supernatants had the organic solvent concentration reduced by adding 1.5 ml of 0.1% HAc and were then loaded onto preconditioned Bond Elut C18 cartridges (Varian). The cartridges were washed with 1 ml of ACN/0.1% HAc (5∶95, v/v) to remove any polar impurities and then eluted with 2 ml of ACN/0.1% HAc (90∶10, v/v). The eluants were dried and reconstituted in 100 µl of ACN/0.1% HAc (80∶20, v/v), then 10 µl injected onto a liquid chromatography-mass spectrometry system for analysis. Appropriate standard curves were prepared similarly with mouse plasma over a concentration range of 0.010 to 20 µg/ml. Liquid chromatography-mass spectrometry was conducted with gradient elution at 0.3 ml/min and detection performed with a single quadrupole instrument fitted with electrospray ionization run in the negative ion mode. Bevirimat was measured at a m/z of 583.5 and the internal standard, DSD, at 585.5.

### Statistical Analysis

Results are expressed as the mean±SEM for each mouse group. Nonparametric statistical analyses were performed by use of the Mann-Whitney U test (StatView 5.0, Abacus Concepts). Data for mice in each group were compared to those for untreated infected mice, and *p*-values ≤0.050 were considered statistically significant.

## Results

Bevirimat Potently Inhibits HIV-1 Replication and Prevents Thymocyte Depletion In Vivo

**Figure 1:**
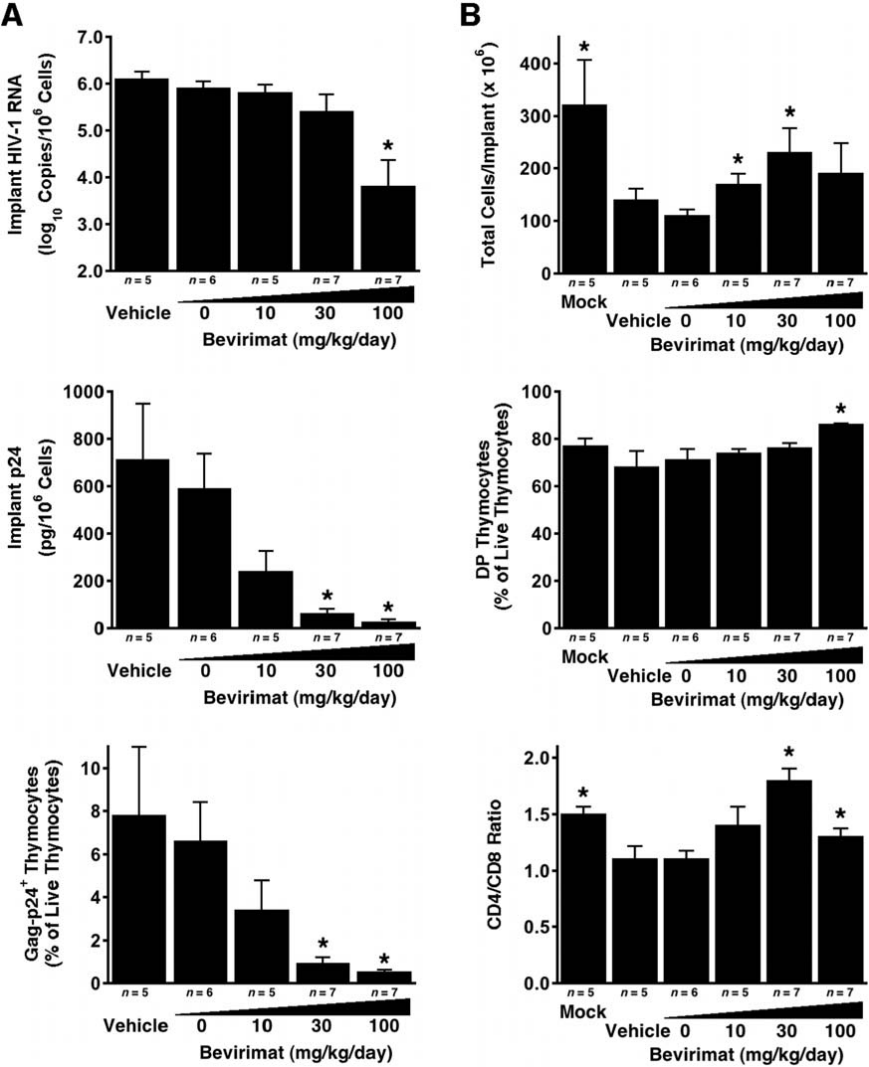
Bevirimat Potently Inhibits HIV-1 Replication and Protects Thymocytes from Virus-Mediated Depletion in Vivo. SCID-hu Thy/Liv mice were treated with bevirimat by twice-daily oral gavage beginning 1 day before inoculation of implants with HIV-1 NL4-3, and dosing was continued until implant collection 21 days after inoculation. Antiviral efficacy was assessed by determining implant viral load (A) and protection from thymocyte depletion (B) for bevirimat-treated mice and mice treated with 10% hydroxypropyl-β-cyclodextrin vehicle alone. Mock-infected mice were not treated. Data are expressed as means±SEM; *p≤0.05 for bevirimat- or vehicle-treated mice versus untreated mice by the Mann-Whitney U test.

We first evaluated the activity of bevirimat against the HIV-1 molecular clone NL4-3 in SCID-hu Thy/Liv mice treated with 10, 30, and 100 mg/kg per day by twice-daily oral administration beginning 1 day before inoculation by direct injection of the Thy/Liv implants and continuing until implant collection 21 days after inoculation. Treatment with bevirimat inhibited viral replication in a dose-dependent manner, reducing implant HIV-1 RNA by 2.1 log_10_ copies, p24 by 96% (590 pg versus 24 pg per 10^6^ cells), and Gag-p24^+^ thymocytes from 7% to <1% at 100 mg/kg per day when compared with untreated mice (Figure 1A). Importantly, bevirimat treatment caused statistically significant protection of the implants from virus-mediated loss of cellularity at 10 and 30 mg/kg per day, from depletion of CD4^+^CD8^+^ (double positive, DP) thymocytes at 100 mg/kg per day, and from a reduction in CD4/CD8 ratio at 30 and 100 mg/kg per day (Figure 1B). Treatment with the 10% hydroxypropyl-β-cyclodextrin vehicle alone had no effect on either viral load or thymocyte depletion in this model.

### Mice Have High Concentrations of Bevirimat in Plasma 0.5–1 h after Oral Administration

**Figure 2:**
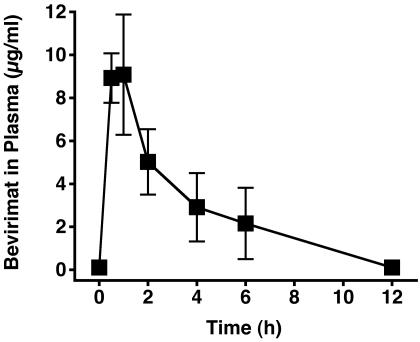
Mice have High Concentrations of Bevirimat in Plasma 0.5–1 h after Oral Administration. Serial plasma samples were collected after the final dose from SCID-hu Thy/Liv mice that had been treated with 100 mg/kg per day for 21 days. Mean±SEM for four mice.

We measured bevirimat concentrations by high-pressure liquid chromatography-mass spectrometry in plasma from uninfected SCID-hu Thy/Liv mice from which serial blood samples were obtained after the final dose (50 mg/kg) following twice-daily treatment with 100 mg/kg per day of bevirimat for 21 days. Peak concentrations of 9 µg/ml (15 µM) bevirimat were attained in plasma 0.5–1 h after oral administration, demonstrating that the N-methylglucamine di-salt of bevirimat is orally bioavailable and capable of achieving peak concentrations 1,500 times higher than the in vitro anti-HIV-1 IC_50_ of 10 nM at this dosage level (Figure 2).

We collected plasma from mice that had been treated for 21 days to assess steady-state concentrations of the drug. The peak levels we observed are consistent with what has been reported for humans after either a single dose [24], [25] or once-daily dosing for 10 days [26]. Bevirimat is mainly eliminated by glucuronidation in rats and humans [24], [27]. Despite the short half-life of about 2 h in the mouse, Figure 2 shows that mean plasma concentrations of bevirimat were >2 µg/ml (>3.4 µM) for 6 h after dosing, and these concentrations are achievable in humans after oral dosing [24]–[26].

### Postexposure Bevirimat Dosing also Potently Inhibits HIV-1 Replication

**Figure 3:**
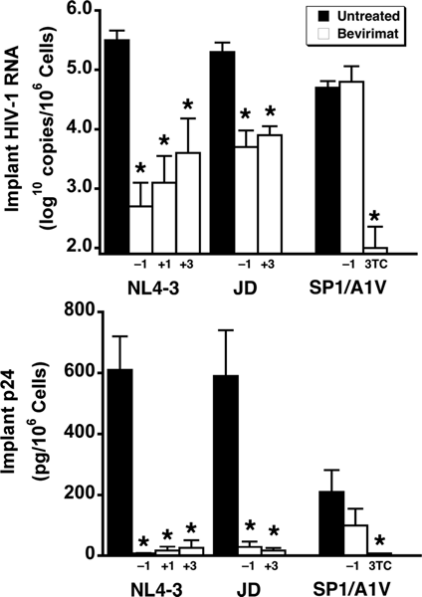
Postexposure Dosing of Bevirimat also Reduces Viral Load. Mice were treated by twice-daily oral gavage with bevirimat at 100 mg/kg per day beginning 1 day before (−1), 1 day after (+1), or 3 days after (+3) virus inoculation of Thy/Liv implants with HIV-1 NL4-3 and JD. Dosing was continued until implant collection 14 days (JD) and 21 days (NL4-3) after inoculation. Antiviral efficacy was assessed by determining cell-associated HIV-1 RNA and p24. There was no reduction in viral load in bevirimat-treated mice inoculated with bevirimat-resistant SP1/A1V, whereas 3TC treatment (30 mg/kg per day by twice-daily oral gavage) was highly effective. Data are expressed as means±SEM; *p≤0.05 for treated mice versus untreated mice by the Mann-Whitney U test for 6–8 mice per group.

Antiviral activity was confirmed in a second SCID-hu mouse study with NL4-3 in which initiation of bevirimat dosing was delayed up to 3 days after virus inoculation as well as in a third study with the AZT-resistant clinical isolate JD (Figure 3). While there was some loss of antiviral potency with delayed dosing initiation, viral load reductions remained >1.0 log_10_ in all treated groups, including the AZT-resistant isolate. To confirm the target of action of bevirimat, NL4-3 with an A-to-V substitution at the N-terminal amino acid of SP1, which has been shown to be resistant to bevirimat in vitro (6), was tested in the mouse model. As expected, the SP1/A1V mutant was resistant to bevirimat treatment but fully sensitive to 3TC (Figure 3).

### Bevirimat Inhibits p24 Release from the CA Precursor (p25) in HIV-1-Infected Human Thymocytes

**Figure 4:**
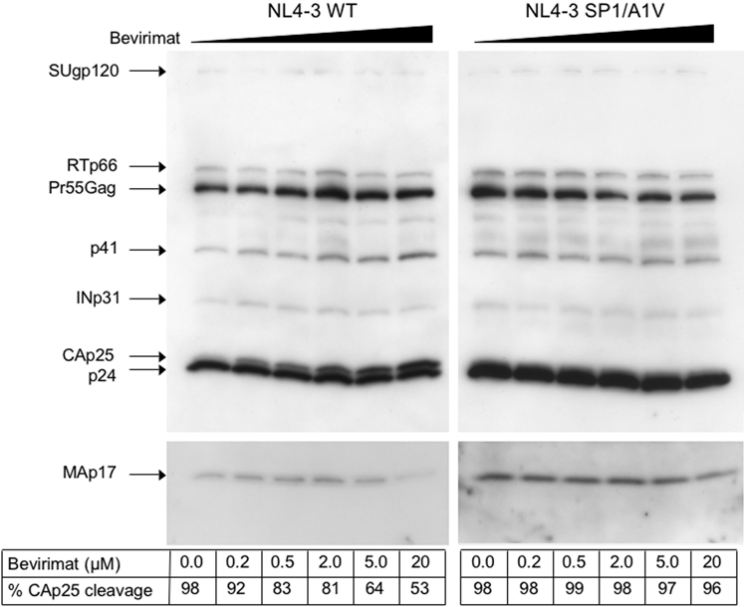
Bevirimat Inhibits p24 Release from the Capsid p25 Precursor in Human Thymocytes. Thymocytes dispersed from NL4-3- and SP1/A1V-infected SCID-hu Thy/Liv implants were cultured in the presence of the indicated range of concentrations of bevirimat for 2 days. Purified virions was lysed and individual viral proteins were detected with HIV-1 Ig. Note the target-specific and dose-dependent reduction of CAp25 cleavage in the presence of bevirimat. The presence of the drug does not affect the stoichiometry of the precursor Pr55Gag polyprotein nor does it inhibit cleavage of the matrix protein, MAp17 (lower panel, longer exposure).

The CA precursor protein (p25) includes a 14-amino-acid SP1 spacer peptide that separates CA from nucleocapsid. It has been convincingly shown in vitro that bevirimat inhibits the cleavage of p25 (CA-SP1) to p24 (CA), leading to a dose-dependent accumulation of p25 in both cell and virion fractions of HIV-1-transfected HeLa cells [2]. We asked whether this was also true for HIV-1-infected implant thymocytes collected from SCID-hu Thy/Liv mice and cultured ex vivo in the presence of bevirimat. Consistent with earlier data, bevirimat specifically disrupted the maturation of CA in virus purified from supernatants of HIV-1-infected human thymocytes, leading to an accumulation of p25 in virions in a dose-dependent manner (Figure 4). Although changes in the levels of MAp17 and p41 were seen 5 and 20 µM, these doses are 30–100 times higher than the concentration required for impairment of CA-SP1 cleavage (0.2 µM) and were likely the result of cytotoxicity. The specificity of bevirimat for CA-SP1 cleavage is also supported by the lack of any obvious treatment effects on the levels of virion reverse transcriptase (RTp66) and integrase (INp31) and the lack of uncleaved NC-SP2-p6 at 15 kDa.

### The A1V Substitution in SP1 Does Not Affect HIV-1 Replication Capacity in the SCID-hu Thy/Liv Mouse Model

Despite the lack of evidence in Figure 4 and in ref. [2] that cleavage of the CA precusor (p25) to mature CA is significantly perturbed in bevirimat-resistant HIV-1 NL4-3 SP1/A1V, it is possible that this CA-SP1 cleavage site mutation could still affect replication capacity as a result of more subtle defects in CA maturation. Indeed, the A-to-V substitution at the N-terminus of SP1 has been reported to cause a twofold decrease in virus production in transfected COS-7 and HeLa cells [28], and Sakalian et al. [29] recently reported more rapid cleavage of CA-SP1 as a possible mechanism for resistance of SP1/A1V in an in vitro particle assembly system. In this assay, the Gag protein forms immature particle structures, although additional mutations at the cleavage site and within SP1 suggested that resistance does not strictly correlate with the rate of cleavage. The more rapid processing of CA-SP1 in the SP1/A1V mutant was confirmed in pulse-chase experiments by Adamson et al. [15], who also showed the extent of CA-SP1 processing does not correlate with bevirimat resistance.

**Figure 5:**
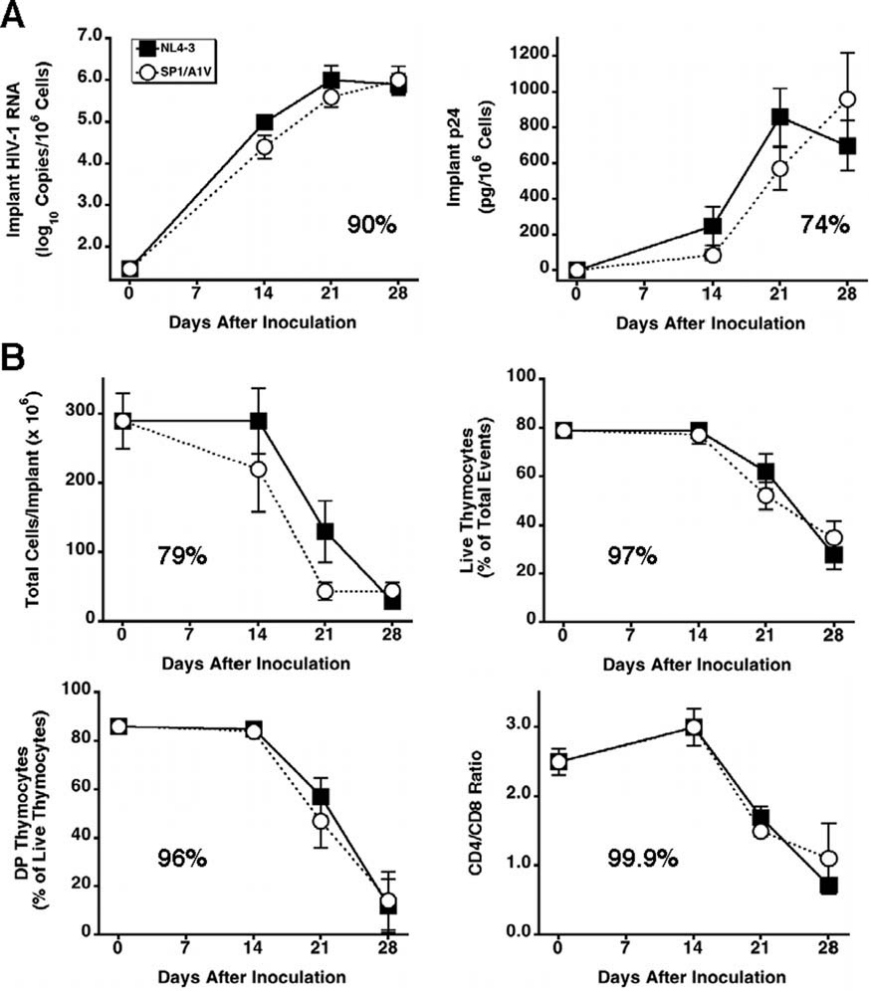
The A1V Mutation in SP1 Does Not Affect Kinetics of Viral Replication or Thymocyte Depletion. Bevirimat-resistant SP1/A1V replicates and depletes thymocytes with kinetics comparable to wild-type NL4-3 in SCID-hu Thy/Liv mice. Viral replication was assessed by determining implant viral load (A), and thymocyte depletion was assessed by total implant cellularity, thymocyte viability, and CD4/CD8 ratio (B) for NL4-3-infected versus SP1/AIV-infected mice for 6 mice per group. Data are expressed as means±SEM; there were no statistically significant differences in viral load or thymocyte depletion at any of the three time points. The number in each graph is the proportion of SP1/A1V to NL4-3 in area under the curve.

**Figure 6:**
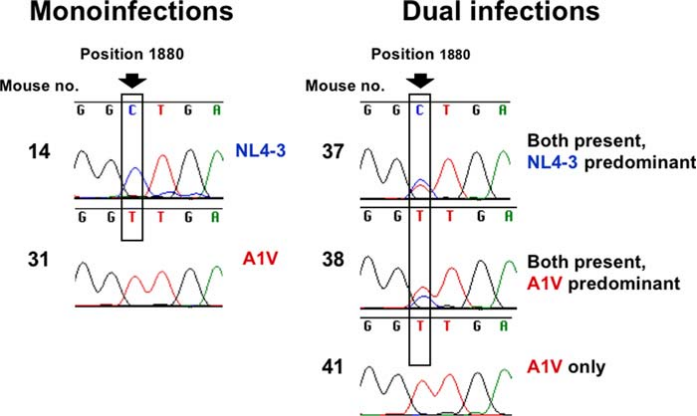
In Vivo Competitions Reveal No Impairment of SP1/A1V Replication. Sequencing of CA-SP1 RNA from three SCID-hu Thy/Liv mice coinfected with equivalent infectious units of NL4-3 and SP1/A1V reveal no impairment of SP1/A1V replication 28 days after implant inoculation. The A-to-V substitution in SP1 is conferred by a C-to-T mutation at nucleotide 1880.

To determine whether bevirimat resistance might confer a fitness disadvantage to the virus in the SCID-hu Thy/Liv mouse model, we compared the replication capacity of SP1/A1V to WT NL4-3 in mice at weekly intervals after inoculation and found no evidence for impaired replication capacity of the SP1/A1V mutant (Figure 5). For both viruses, peak viral loads were attained 21–28 days after inoculation as the implants became severely depleted of thymocytes. We obtained similar time-dependent increases in viral RNA and p24 for both viruses with no statistically significant differences in viral load at any of the three time points (Figure 5A). The results were mirrored by similar thymocyte depletion kinetics in terms of reductions in implant cellularity, thymocyte viability, percentage of DP thymocytes, and CD4/CD8 ratio (Figure 5B). Comparison of NL4-3 and SP1/A1V kinetics by area-under-the curve analysis showed SP1/A1V to have 90% the area-under-the curve of NL4-3 for viral RNA, 74% for p24, and 79–99.9% for the parameters of thymocyte depletion shown in Figure 5B. Further support for the lack of impaired replication capacity of the SP1/A1V mutant was provided by virus competitions performed by coinfecting Thy/Liv implants with a mixture of equivalent infectious units (500 50% tissue-culture infectious doses each) of WT NL4-3 and SP1/A1V and collecting them 21 or 28 days after coinoculation. As shown in Figure 6 and Table 1, both genomes were detected in six of nine coinfected mice by sequencing CA after RNA RT-PCR amplification. In mixing experiments, one genome had to be present at 10–20% of the total for the minor species to be detected as a peak (the A-to-V substitution in SP1 is conferred by a C-to-T mutation at nucleotide 1880) in the sequencing chromatogram. We surmised that the three coinfected samples with only the A1V genome seen in the chromatogram might have WT genome below the level of detection, so we performed colony sequencing to obtain more precise percentages. The clonal analysis showed 8–23% A1V sequences for these three implants, and the percentages for the other implants were in agreement with the relative proportions of the genomes in the chromatograms. For all nine mice, there was a mean of 44% WT and 56% A1V clones. In addition, there was no evidence of reversion to the WT alanine in SP1/A1V-infected implants at day 28.

**Table 1:** In Vivo Competition Results for NL4-3 and Bevirimat-Resistant SP1/A1V.

Mouse no.	p24 (pg per 10^6^ cells)	HIV-1 RNA (log_10_ copies per 10^6^ cells)	Sequences detected in chromatogram (predominant) a	Colony sequences (%)	
				NL4-3	A1V
Expt 1 (28 days)					
37	2,856	6.89	Both (NL4-3)	63	37
38	1,212	6.66	Both (A1V)	49	51
41	1,227	6.53	A1V	7	93
Expt 2 (21 days)					
32	329	5.60	Both (NL4-3)	45	55
33	529	5.81	Both (NL4-3)	70	30
34	201	5.35	A1V	8	92
35	420	5.84	A1V	23	77
36	210	5.12	Both (NL4-3)	67	33
40	215	5.71	Both (NL4-3)	61	39
MEAN (9 mice)				44	56

a Viral species identified by sequencing amplicons generated after RT-PCR of CA RNA as shown in Figure 6.

### Protease Inhibitor-Resistant HIV-1 Is Hypersensitive to Bevirimat

**Figure 7:**
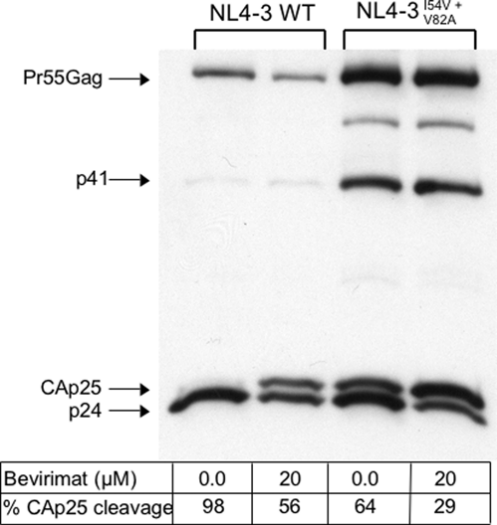
Protease Inhibitor-Resistant HIV-1 has Impaired CA-SP1 Cleavage. Wild-type NL4-3 and protease mutant NL4-3 PR_I54V+V82A_ virions were collected from transfected 293T cells grown in the presence (20 µM) or absence of bevirimat and analyzed for particle maturation by Western blot using a p24 monoclonal antibody. The protease mutant is deficient in CA-SP1 cleavage and is comparable to bevirimat-treated wild-type virus, however, the drug has a more dramatic effect on CA-SP1 cleavage in HIV-1 PR_I54V+V82A_.

**Figure 8:**
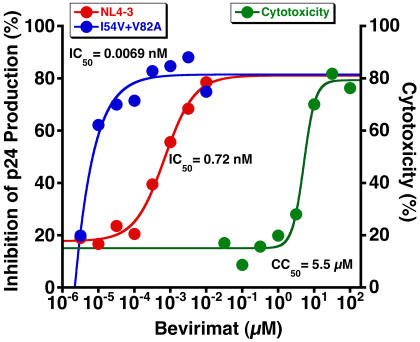
Protease Inhibitor-Resistant HIV-1 with Impaired CA-SP1 Cleavage Is Hypersensitive to Bevirimat In Vitro. NL4-3 PR_I54V+V82A_ is hypersensitive to bevirimat in PHA-activated PBMCs with 50% inhibitory concentration (IC_50_) >100 times lower than for wild-type NL4-3. CC_50_ is the 50% cytotoxic concentration as assessed by MTT assay.

HIV-1 with drug-resistance substitutions in protease display multiple Pr55Gag cleavage defects, in particular at the CA-SP1 junction, resulting from impairment of the catalytic efficiency of the mutant enzyme [30], [31]. We reasoned that this might synergize with bevirimat activity, so we treated WT- and protease-mutant (PR_I54V+V82A_)-transfected 293T cells with bevirimat, and subjected supernatant virions to Western blot analysis of p25 cleavage. As expected, the CA precursor (p25) of NL4-3 PR_I54V+V82A_ was incompletely cleaved (64% cleavage) whereas the p25 of WT NL4-3 was cleaved to completion (Figure 7A). Interestingly, p25 cleavage of NL4-3 PR_I54V+V82A_ was even more impaired in the presence of 20 µM bevirimat (29% cleavage). We anticipated that this might render NL4-3 PR_I54V+V82A_ hypersensitive to bevirimat compared to WT NL4-3, and this was shown to be the case in PHA-activated PBMCs (Figure 8). The mean IC_50_ for NL4-3 PR_I54V+V82A_ was 0.010±0.0032 nM (n = 7; range: 0.0031–0.024), which was 140 times lower than for WT NL4-3 (1.4±0.47 nM; range: 0.18–3.0).

## Discussion

In this study we demonstrate that the HIV-1 maturation inhibitor bevirimat has potent antiviral activity in SCID-hu Thy/Liv mice after oral administration. This is the first report demonstrating in vivo activity of bevirimat and the first time in a number of years that a novel target for HIV-1 inhibition has been identified and validated in an animal model. In HIV-1-infected humans treated with 10 days of oral bevirimat monotherapy, potent suppression of viral replication was observed with a median 1 log_10_ reduction in viral load at the 200-mg dose level (unpublished data). When an adjustment is made for the 12-fold difference in surface area-to-body weight ratio between mice and humans [32], [33], the human dose of 200 mg per day corresponds to 41 mg/kg per day in the mouse. This approximates the 100 mg/kg per day dosage level that we show here to have potent antiviral activity in the SCID-hu Thy/Liv model.

This analysis also confirmed the target specificity of the drug which did not affect the processing of Pr55Gag except at the CA-SP1 junction in infected human thymocytes treated ex vivo. Additionally, bevirimat treatment had no effect on the processing of CA-SP1 in bevirimat-resistant SP1/A1V, as no uncleaved CA precursor (p25) was detected even at a concentration of 20 µM. These results confirm the antiviral mechanism of action of bevirimat and are consistent with the potent inhibition of HIV-1 replication we have observed in the SCID-hu Thy/Liv model.

While the SP1/A1V mutant was shown to be replication competent in this model, it should be noted that the amino acid sequence at the CA-SP1 cleavage junction, including the alanine at position one of SP1, is highly conserved among different HIV-1 strains and to our knowledge no naturally occurring polymorphisms have been reported at this residue. This suggests that there may be selective pressure to maintain this sequence in HIV-1-infected humans, and de novo generation of the SP1/A1V mutant may not readily occur. Further support for this assertion is provided by the lack of resistance development observed in viral population sequencing of clinical samples from patients treated with bevirimat in a 10-day monotherapy study (unpublished observations) where there was continued drug exposure for approximately three weeks as a result of bevirimat's long half-life of 2.5–3 days [26]. Nevertheless, the fact that the SP1/A1V mutant is replication competent in this model does suggest that continued screening for this mutation in the clinical program is warranted. Single mutations in reverse transcriptase (RT) confer resistance to nucleoside RT inhibitors (M184V, K65R) and nonnucleoside RT inhibitors (Y181C) without significantly compromising viral fitness, yet these drugs are used in most antiretroviral regimens. The standard of care for drug-naïve patients is to treat with potent combination therapy regardless of the specific potency of any of the drugs in the combination.

Our observation that protease-inhibitor resistant NL4-3 PR_I54V+V82A_ with impaired CA-SP1 cleavage is hypersensitive to bevirimat activity represents a unique form of antiviral synergy in which a drug-resistant enzyme inefficiently cleaves the same site where a drug exerts its antiviral activity. This observation has important clinical relevance because it might be predicted that patients with protease-inhibitor resistance mutations that impair CA-SP1 cleavage (such as PR_I54V+V82A_) might respond to bevirimat treatment with greater viral load reductions than drug-naïve patients with WT HIV-1. It is not feasible to study protease-inhibitor-resistant HIV-1 in the SCID-hu Thy/Liv model because replication rates of the resistant mutants are not comparable to the rates in other systems [20]. Replication of HIV-1 PR_I54V+V82A_ occurs reproducibly in PHA-activated PBMCs, so in vitro antiviral assays were performed with these cells.

The SCID-hu Thy/Liv studies presented here showed potent suppression of HIV-1 replication by bevirimat whether it was administered before or up to three days after inoculation, and potent suppression was also observed against an AZT-resistant clinical isolate. Because drug resistance, and particularly cross-resistance, is a significant problem in antiretroviral therapy, it is notable that we were able to demonstrate that 3TC was highly effective against bevirimat-resistant SP1/A1V.

The efficacy of bevirimat observed in SCID-hu Thy/Liv mice clearly establishes the proof-of-concept for this novel inhibitor of HIV-1 replication. Currently, all approved small molecule, orally bioavailable inhibitors of HIV-1 replication target one of two HIV-1 viral enzymes: reverse transcriptase or protease. Small-molecule antiretroviral drugs in development include agents targeting the HIV-1 integrase enzyme or the viral coreceptors, CCR5 or CXCR4. In contrast, bevirimat is a small orally bioavailable molecule that does not inhibit HIV-1 replication through a viral enzyme or receptor, thus providing a uniquely high target specificity with the potential for minimal adverse affects on cellular pathways.

Taken together, these results establish bevirimat as a potent inhibitor of HIV replication in vivo and confirm the success of the SCID-hu Thy/Liv model as a valuable predictor of clinical utility. In short-term (i.e., 10-day) human studies, bevirimat was well tolerated and had pharmacokinetics supporting once daily or even less frequent oral dosing [24]–[26]. These results support further clinical development of this first-in-class maturation inhibitor and demonstrate the usefulness of the SCID-hu Thy/Liv model for evaluating in vivo antiretroviral efficacy, drug resistance, and viral fitness, particularly for novel targets of antiviral inhibition.
